# Intelligent Rapid Adaptive Offloading Algorithm for Computational Services in Dynamic Internet of Things System

**DOI:** 10.3390/s19153423

**Published:** 2019-08-04

**Authors:** Xuejing Li, Yajuan Qin, Huachun Zhou, Yongtao Cheng, Zhewei Zhang, Zhengyang Ai

**Affiliations:** School of Electronic and Information Engineering, Beijing Jiaotong University, Beijing 100044, China

**Keywords:** deep neural network, edge computing, Internet of things, offloading policy, resource allocation

## Abstract

As restricted resources have seriously limited the computational performance of massive Internet of things (IoT) devices, better processing capability is urgently required. As an innovative technology, multi-access edge computing can provide cloudlet capabilities by offloading computation-intensive services from devices to a nearby edge server. This paper proposes an intelligent rapid adaptive offloading (IRAO) algorithm for a dynamic IoT system to increase overall computational performance and simultaneously keep the fairness of multiple participants, which can achieve agile centralized control and solve the joint optimization problems related to offloading policy and resource allocation. For reducing algorithm execution time, we apply machine learning methods and construct an adaptive learning-based framework consisting of offloading decision-making, radio resource slicing and algorithm parameters updating. In particular, the offloading policy can be rapidly derived from an estimation algorithm based on a deep neural network, which uses an experience replay training method to improve model accuracy and adopts an asynchronous sampling trick to enhance training convergence performance. Extensive simulations with different parameters are conducted to maintain the trade-off between accuracy and efficiency of the IRAO algorithm. Compared with other candidates, the results illustrate that the IRAO algorithm can achieve superior performance in terms of scalability, effectiveness and efficiency.

## 1. Introduction

Along with the development of information and communication technology, a large number of wireless devices urgently require better processing capability for computation-intensive services [1]. Meanwhile, the computational performance of sensor nodes and Internet of things (IoT) terminals is seriously limited by the restricted resources in computing, caching and energy. Consequently, service migration has been proposed as an innovative technique of cloud-network convergence, which can selectively offload services from the terminals to cloud servers with powerful capability [2]. It is constrained by the limited radio resource in radio access network (RAN) to offload massive data from wireless devices and support real-time services. With many technologies, such as beam forming and carrier aggregation, the RAN is evolving in terms of radio resource and can provide larger bandwidth, which makes the service migration a more promising solution [3]. However, on account of the long distance between the terminals and the servers located at a core network in cloud computing, there are some challenges relating to additional bandwidth consumption, longer propagation latency and extra security threats. Therefore, mobile edge computing (MEC) has emerged as an enabling technology which promotes edge-cloud collaboration by deploying servers in close proximity to the user terminals. In other words, quality of service (QoS) can be further improved by offloading the services to nearby servers located at the edge of the RAN [2]. 

In MEC-based networks, it is complicated to design offloading policy for a dynamic IoT system. Adaptive policies have to be made continually due to the rapid dynamicity of wireless channels, device capacities and service requirements. In ultra-dense networks, heterogeneous accessed devices further complicate the design of offloading policy from a systematic perspective. Also, it is more crucial to allocate the radio resource for different participants at a wireless access point (AP) or base station (BS), on account of that the computational capability and storage capacity are powerful and virtualized at the side of an MEC cloud [3]. Thus, a joint optimization problem arises related to offloading policy design and radio resource allocation, which is so complicated that it cannot be solved adaptively and rapidly by conventional iterated-based optimization algorithms. 

In this paper, we consider a time-varying IoT system consisting of multiple IoT devices and one AP deployed in an MEC network. Constrained with respective limited capacities of IoT devices [4], they have diverse computational requirements and different wireless channels accessed with the AP. In order to increase the overall system performance and simultaneously keep the fairness of different participants, a joint optimization problem is formulated to maximize the utility calculated by the weighted sum computing rate of all users. We propose an intelligent rapid adaptive offloading (IRAO) algorithm for a dynamic IoT system to realize agile centralized control from the view of wireless AP. Unlike other conventional algorithms, the IRAO algorithm can continually solve the joint optimization problem by rapidly deriving adaptive combinatorial strategies related to offloading policy design and radio resource allocation. For reducing the execution time of the algorithm, several machine learning methods are applied and a learning based framework is constructed by three parts—offloading decision-making, radio resource slicing and algorithm parameters updating. Firstly, offloading policies are derived from an estimation algorithm based on a deep neural network (DNN) model. Secondly, radio resource is allocated by slicing technique with an iterated based optimization method. Thirdly, we exploit an experience replay training method to improve the DNN model accuracy. To further accelerate the training convergence rate and reduce the possible of over-fitting, asynchronous and important sampling tricks are applied in the training method. 

The main contributions of this paper are summarized as follows. First, a joint optimization problem is formulated to maximize the system utility with comprehensive considerations of a heterogeneous system including wireless channels, device capacities and service requirements. Second, to be more realistic, we consider a time-varying system. A learning based algorithm named IRAO is designed for a dynamic IoT system to rapidly derive adaptive combinatorial strategies. To make suitable offloading decisions, an estimation algorithm is designed based DNN with low computation complexity. Third, we find suitable parameters of the IRAO algorithm and modify the training process of DNN. Compared with other benchmark algorithms, the IRAO algorithm can derive near-optimal strategies rapidly and guarantee good performance in large-scale networks. 

The rest of this paper is organized as follows. In Section 2, some related works are reviewed. In Section 3, we introduce the dynamic system model and formulate the optimization problem. In Section 4, the IRAO algorithm is presented and three DNN-based models are designed. In Section 5, numerical results are illustrated and discussed. Finally, this paper is concluded in Section 6.

## 2. Related Works

In this section, we review numerous works of research which focus on offloading policy decision, radio resource allocation and intelligent algorithm application.

### 2.1. Offloading Policy and Radio Resource

With the technology of software-defined radio and cognitive radio, the resources in RAN can be managed and allocated flexibly [5,6]. Qian et al. [7] solved a joint base station association and power control optimization problem to maximize the system utility and minimize the transmit power based on game theory. The corresponding strategies were adaptive and customized due to the dynamicity and diversity of wireless channel states in the RAN. In reference [8], a non-convex nonlinear programming problem was formulated and decomposed into two convex sub-problems which are related to the transmit power allocation of base station and the backhauling bandwidth allocation from cell networks to BS. To maximize the energy efficiency under power constraint and data rate requirements, a near-optimal iterative algorithm and a sub-optimal low-complexity algorithm were designed for a system based on the frequency division multiple access (FDMA) technique, which is able to divide the frequency spectrum into several small-grained bands for different accessed users. Nevertheless, references [7,8] did not consider the offloading policy to decide whether to offload services or not. Chen et al. [9] researched a multi-user computation offloading problem in a multi-channel wireless interference environment and proposed a distributed algorithm based on game theory since it is NP-hard to compute a centralized optimal solution. This work only considered that the power is determined to satisfy the requirements of wireless transmission and it can be extended to a joint optimization problem combined with the power control. In reference [10], a problem was formulated as a mixed integer nonlinear program (MINLP) that involves jointly optimizing the task offloading decision, uplink transmission power of mobile users and computing resource allocation at the MEC servers. It was studied to maximize the users’ task offloading gains measured by weighted sum reductions in task completion time and energy consumption. The resource allocation problem was addressed by convex optimization techniques based on a bi-section search method and the task offloading problem was solved by a novel heuristic algorithm with suboptimal performance. However, references [9,10] did not consider the radio resource allocation problem.

In an IoT system based on MEC networks, the offloading policy highly depends on the channel quality of wireless data transmission and many researchers [11,12,13,14,15,16,17,18] investigated the joint optimization problems related to offloading policy and radio resource. In a multi-mobile-users MEC system, the authors [11] formulated an MINLP problem to minimize energy consumption, subjected to specific application latency constraints. A reformulation-linearization-technique-based Branch-and-Bound method is proposed to obtain optimal results and a Gini coefficient-based greedy heuristic is designed to degrade the complexity. Besides the FDMA technique, You et al. [12] considered a multi-user system based on the time division multiple access (TDMA) technique, which enables several users to share same frequency spectrum by dividing transmitting data into different time slots. With infinite or finite cloud computation capacity, the optimal resource allocation was formulated as a convex optimization problem for minimizing the weighted sum mobile energy consumption under the constraint on computation latency. An offloading priority function was defined to yield priorities for users, which depends on their channel qualities and local computing energy consumption. Based on this, a threshold-based bisection search algorithm was proposed for generating offloading policy. And a sub-optimal algorithm was proposed with a more effective function based on approximated offloading priority to reduce the complexity arising from a two-dimensional search for Lagrange multipliers. In reference [13], the energy-efficient resource-management policy was further studied for an asynchronous system where the mobiles have heterogeneous input-data arrival time instants and computation-deadline constraints. Related to data partitioning for offloading and time division for transmissions, a joint optimization problem was formulated to minimize the total mobile-energy consumption. The authors extended the threshold-based bisection search algorithm and used the block coordinate descent (CD) optimization method based on an iterative searching structure. With an energy harvesting (EH) technique that can provide sustainable operation for sensor devices, the energy-limited devices can avoid the service disruptions caused by manual battery replacement or recharging [14,15,16]. Based on the EH model in a single-user system and the task deadline constraints, a framework was proposed in reference [14] for energy-efficient computing with some strategies for local processor cycles controlling, radio resource division and mode selection. By contrast, reference [15] considered a multi-user system based on TDMA. For minimizing the total energy consumption subject to the individual computation latency constraints, a resource allocation scheme was developed related to energy transmitting, frequency control and time allocation. To maximize the sum computation rate of all wireless devices, reference [16], proposed a joint optimization problem related to individual computing offloading decisions and transmission time allocation, which is solved by two decoupled problems with a bisection search algorithm and a CD method. An alternating direction method of multipliers was proposed to reduce the high computational complexity of the CD method in a large-size network. In references [17,18], an energy-efficient dynamic offloading and resource scheduling policy was provided and extended by relaxing the binary constraints to variables, which was combined with clock frequency control and transmission power allocation which was extended. However, those iterative-based methods all had high computational complexity to reach a satisfying local optimum, which is still not appropriate for fine-grained control in dynamic large-scale networks. 

### 2.2. Intelligent Algorithm Application

To cope with the problem in large-scale networks which are rapidly changing over time, the adaptive fine-grained control of resource allocation and offloading decisions becomes more challenging. Applied to artificial intelligence (AI) and machine learning (ML) algorithms, the complicated problems can be solved while guaranteeing both efficiency and optimality. As an important branch of ML, deep learning (DL) based algorithms specialize in approximating the input-output non-linear mapping relationship to solve expensive computational problems [19,20]. Extended from DNN, convolutional neural network (CNN) is applied to compress the input values and improve algorithm performance. Supervised learning algorithm can achieve data classification and prediction by a training process with manually labeled samples. To solve the problems about dynamic programming and Markov decision process (MDP), it is effective to use learning-based algorithms without the external supervisor to minimize long-term cost, such as increment learning and reinforcement learning (RL) [21,22]. With a learning-based structure, rational solutions can be obtained after sufficient training episodes with successive input values. Further, Q-learning is a simple RL-based algorithm with tabular-search nature, which is not suitable for handling dynamic problems with high dimensional space. To improve the algorithm efficiency, deep Q-network (DQN) is one kind of deep RL (DRL) based algorithms combined with DL technique. Reference [23] presented a comprehensive survey on deep learning applied in mobile and wireless networking. In reference [24], learning-based approaches were proposed for the radio resource interference management and wireless transmitting power allocation. Xu et al. [25] designed a novel DRL-based framework for power-efficient resource allocation while meeting the demands of wireless users in highly dynamic cloud RANs. 

There are also several pieces of researches which apply these intelligent algorithms to offloading decision-making in MEC networks. In reference [26], a double dueling DQN based algorithm was proposed to enable dynamic orchestration of networking, caching and computing resources. It improved the performance of applications but did not consider the energy efficiency issue. Based on CNN and RL, reference [27] presented an offloading scheme for an individual IoT device with EH to select edge server and offloading rate according to current battery level, previous radio transmission rate and predicted harvested energy. Simulation results showed that the scheme can reduce the energy consumption, computation latency and task drop rate. Similar to reference [27], which only considered a single device, reference [28] added a task queue state model to dynamic statistics as well as a channel qualities model and an energy queue state model. A double DQN based computation offloading algorithm was proposed for a single user device as well. Considering a joint optimization problem, a parallel DNN model was designed in reference [29] to make binary offloading decisions and bandwidth allocation decisions. For minimizing the utility calculated by energy consumption and task completion delay, the algorithm only considered the different computation amount of tasks in several devices without the dynamic wireless channel state. However, all these learning based algorithms have the convergence performance problem which is caused by over-fitting training nature of simple training methods. This paper proposes a learning-based algorithm named IRAO which applies a DNN-based estimation algorithm with asynchronous experience training method. Table 1 is presented for a quick sight of the main characteristics of different related works and our work.

## 3. System Modeling and Problem Formulation

In this section, we introduce a dynamic IoT system model, a local computing mode and an offloading computing mode. The optimization problems are formulated at last.

### 3.1. System Model

**Figure 1:**
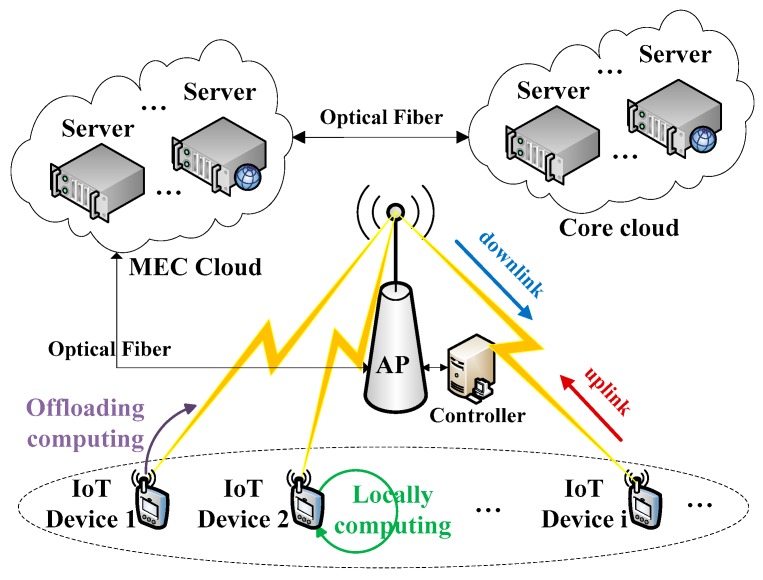
The system model with multiple devices and dynamic channels.

As shown in Figure 1, we consider an IoT system with multiple IoT devices, one wireless AP, one software-defined controller attached to the AP and virtualized servers in MEC cloud. These devices belonging to respective users are assumed to be heterogeneous and are represented by I={1, 2, … , I}. We ignored the communication latency between the server, the controller and the AP, because they are all deployed closely and connected with optical fiber in an MEC network. With network functions virtualization (NFV) technology which enables operators to virtualize and orchestrate resources of computation, cache and network, we assume that there are sufficient virtualized resources at the side of an MEC cloud. On each device i∈I, there are dynamic continuous computational tasks which are delay-sensitive. The energy resource on each device is denoted by $E_{i}$ and is constrained by the current energy and harvest energy. As many techniques are widely studied for energy harvesting [30], we assume that the devices can harvest continuous energy over time. In order to achieve the fine-gained control, we divide the system time into sequential time frames of identical duration denoted by T. During each current time frame, the device is assumed to use up the energy $E_{i}$ which is harvested in the previous time frame. It is denoted by $E_{i}=\mu_{i}T$, where $\mu_{i}$ denotes the energy harvesting efficiency coefficient The wireless channel power gains are used to indicate the channel quality of data communicating, which are considered to be the same in uplink and downlink and generally caused by several factors, including path loss, shadowing and fading. Correspondingly, we use $G_{i}$ to denote the channel power gain between each device i and the AP. In order to realize the fine-grained adaptive control of the dynamic system, we divide the continuous time into periodic time frames which are equal and short. The dynamic channel power gain is discretized as some different quasi-static values in each time frame. Accordingly, we assume that the channel power gains remain unchanged during each short-term frame but vary across different frames. 

For avoiding mutual communication interference in a wireless channel, we consider the TDMA technique which is usually applied to allocate radio resource under the same frequency band. Based on the programmable air interface, each time frame T is assumed to be divided into three phases for different purposes and further divided into several slices for different user devices. At the first phase of each time frame, the AP needs to communicate with the devices to transmit signaling traffic for synchronizing and controlling information, such as wireless channel power gains, computational service demand levels, available device energy resources and corresponding intelligent control strategies. We assume that this phase is completed in a short duration denoted by 𝒶T where 𝒶≪1. With the software-defined networking (SDN) technology which enables the separation of control plane and data plane, the signaling traffic and the data traffic can be transmitted on the two different planes respectively without interfering with each other. Thus, this phase is assumed to be accomplished on the control plane without occupying the radio resource of the data plane, which is called out-band controlling. Following the first phase, we should allocate several time slots for data offloading in the second phase. The allocated slice is denoted as $\tau_{i}T$ for each user device i, where $\tau_{i}$ is constrained by $\tau_{i}\in \left[0,1\right]$. Particularly, we do not allocate slice for device i in this phase if $\tau_{i}=0$, that is, the device is in a local computing mode which is detailed in the next subsection. Because the related states in the system environment vary across time frames, the radio resource slicing scheme in the second phase needs to be adaptive. For the reason that the computing speed of the MEC server is much faster than the devices constrained with limited resources, the computing time spent on the server can be negligible [12]. In the last phase, the AP requires some time for returning computing results to respective devices. Because the transmit power of the AP is large enough and the downloading data size in downlink is much less than the offloading data in uplink [12], the slice allocated for data downloading is set as a small value denoted by $\lambda_{i}T$. With the above-mentioned assumptions, the frame slicing parameters $\tau_{i}$ and $\lambda_{i}$ are constrained as follows:

(1) $$\sum_{i=1}^{N}\tau_{i}+\lambda_{i}\leq 1$$

In the system, we consider a classical binary offloading policy in which the computational service is either offloaded or executed locally [2]. The computing offloading decision variable is denoted by ${𝓍}_{i}\in \left\{0,\;1\right\}$. The computational service of user i is executed locally at the device itself if ${𝓍}_{i}=0$, that is, the device i is in a local computing mode. Meanwhile, The computational service of user i is offloaded to the server if ${𝓍}_{i}=1$, that is, the device i is in an offloading computing mode.

### 3.2. Local Computing Mode

In the local computing mode, the computational service is executed locally at the device itself with constrained computation capability. We denote the processor computation clock frequency in user device i as $f_{i}$ in the unit of cycles per second. Let  ∅>0 denote the number of cycles needed to process one data bit. With the service completion time constrained by $0\leq t_{i}\leq T$, the local computational service workload could be denoted by $L_{l,i}=f_{i}t_{i}$ and the processed bits could be expressed by $b_{l,i}=f_{i}t_{i}/\emptyset$. In addition, the energy consumption of the device i is constrained by $E_{l,i}=e_{i}L_{l,i}f_{i}^{2}=e_{i}f_{i}^{3}t_{i}\leq E_{i}$, where $e_{i}$ denotes the computation energy efficiency coefficient depending on the chip architecture [17]. In order to reach a faster computing rate with less energy consumption in each device, it is obvious to infer that the service computing time should be larger and the frequency should be smaller, which is based on the dynamic voltage and frequency scale method [7,8]. Thus, we assume that the service computing time continues throughout the whole time frame T, that is, $t_{i}^{*}=T$. Because the computational capability is constrained by the max computation clock frequency of the processor, which is denoted by $f_{i,max}$, we assume that the energy $E_{i}$ is so low that the executed clock frequency $f_{i}$ would not reach the max clock frequency $f_{i,max}$. Accordingly, the optimal clock frequency is given by $f_{i}^{*}={\left(E_{i}/e_{i}T\right)}^{1/3}$ when the energy $E_{i}$ is exhausted during the computing process. With $e_{i},\emptyset$ denoting the static physical parameters of the device, the maximum local computing rate in the unit of bits per second is given by 

(2) $$r_{l,i}^{*}\left(\mu_{i}\right)=\left(f_{i}^{*}t_{i}^{*}\right)/\left(\emptyset T\right)={\left(\mu_{i}/e_{i}\right)}^{1/3}/\emptyset$$

### 3.3. Offloading Computing Mode

In the offloading computing mode, the service is offloaded to the MEC server through dynamic wireless channel. As the computing capability of server is powerful based on NFV, we assume the computing time consumed in this mode is negligible. In the scenario of a low end-to-end latency network, the offloading computing rate is mainly constrained by the information transfer rate. As the Shannon Hartley equation is given by $C=W\log_{2}\left(1+S/N\right)$ where *W* denotes the channel bandwidth and *N* denotes channel noise power, it can be inferred that the maximum information transfer rate of the channel C is larger if the transmitting signal power S is bigger. Thus, we assume the signal power of device i to be set to the maximum value calculated by $S_{i}=(E_{i}/\tau_{i}T)*G_{i}$ and the available energy $E_{i}$ is assumed to be used up during the frame slice $\tau_{i}T$. Therein, the energy consumed on the IoT devices for downloading data is negligible, because the transmit power of the AP is large enough and the downloading data in downlink are much smaller. Then the maximum offloading computing rate is assumed to be equal to its data offloading rate which is given by

(3) $$r_{o,i}^{*}\left(\tau_{i}\right)=(W\tau_{i}/v_{u})\log_{2}\left(1+\mu_{i}\;G_{i}/\tau_{i}N\right)$$

where $v_{u}$ denotes the bandwidth utility ratio coefficient, which depends on the signaling load of message segments in the data traffic.

### 3.4. Problem Formulation

We discretize the dynamic channel power gains as individual sequential sets with quasi-static values in each time frame, that is, $G=\{G_{i}|i\in I\}$. Meanwhile, we use $\Omega =\{\Omega_{i}|i\in I\}$ to represent the set of computational service demand levels in each time frame which are quantized by dynamic service requirements and weight $\Omega_{i}$ denotes each level of computational service in device i. Besides, $E=\{E_{i}|i\in I\}$ denotes the set of available energy on device i. To improve the overall system computational performance and simultaneously maintain the fairness for different participants, we define an optimization problem to get the maximum utility calculated by weighted sum computing rate as

(4) $$U\left(G,\Omega ,E,𝓍,\tau \right)≜\overset{I}{\underset{i=1}{\sum }}\Omega_{i}\left(\left(1-{𝓍}_{i}\right)r_{l,i}^{*}\left(\mu_{i}\right)+{𝓍}_{i}r_{o,i}^{*}\left(\tau_{i}\right)\right)$$

In this case, $𝔁=\{{𝓍}_{i}|i\in I\}$ represents the set of offloading decisions and $\tau =\{\tau_{i}|i\in I\}$ represents the set of radio resource slices. We formulate the first problem as U1(G,Ω,E):

(5) $$U1^{*}\left(G,\Omega ,E\right)=\underset{𝓍,\tau }{max}\;U\left(G,\Omega ,E,𝔁,\tau \right)$$

$$s.t.C1:\sum_{i=1}^{I}\tau_{i}+\lambda_{i}\leq 1$$

$$C2:\tau_{i}\in \left[0,1\right]$$

$$C3:\lambda_{i}\in \left[0,1\right]$$

$$C4:{𝓍}_{i}\in \{0,1\}.$$

However, this problem is difficult to solve because it is a mixed integer programming non-convex problem. For reducing the complexity, we divide the problem into two sub-problems namely, the offloading decision-making problem and the time frame slicing problem. First, the computing offloading decisions problem requires to find an optimal or suboptimal decision 𝔁 among all $2^{I}$ possible computing offloading decisions, that is, $𝔁\in {\left\{0,\;1\right\}}^{I}$, which are derived by iteratively swapping offloading decision ${𝓍}_{i}$ of each user device. The search space increases exponentially with the number of users I if we use the exhaustive or heuristic methods to find the optimal decision [16]. Applied some machine learning algorithms which could reduce the execution time of the algorithm [27,29], the decisions could be made in real-time under quickly varying conditions. We formulate the second problem as $U2^{*}\left(G,\Omega ,E\right)$:

(6) $$U2^{*}\left(G,\Omega ,E\right)=\underset{𝓍}{max}\;U\left(G,\Omega ,E,𝔁\right)$$

$$s.t.C4:{𝓍}_{i}\in \{0,1\}.$$

Second, when the binary offloading decisions 𝔁 are given, we need to solve the time frame slicing problem which is a convex problem of allocating the optimal frame slice $\tau^{*}$. We apply a one-dimensional bi-section search algorithm which was introduced in paper [12]. And we formulate the third problem as $U3^{*}\left(G,\Omega ,E,𝔁\right)$,

(7) $$U3^{*}\left(G,\Omega ,E,𝔁\right)=\underset{\tau }{max}\;U\left(G,\Omega ,E,𝔁,\tau \right)$$

$$s.t.C1:\sum_{i=1}^{I}\tau_{i}+\lambda_{i}\leq 1$$

$$C2:\tau_{i}\in \left[0,1\right]$$

$$C3:\lambda_{i}\in [0,1].$$

## 4. Intelligent Rapid Adaptive Offloading Algorithm

In this section, we design an IRAO algorithm for a system controller which has knowledge of channel quality and user requirements.

### 4.1. Algorithm Framework

**Figure 2:**
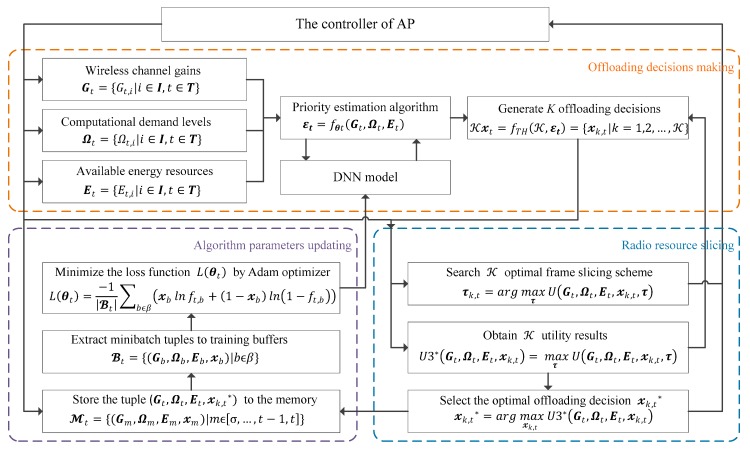
Iterated learning-based framework of intelligent rapid adaptive offloading (IRAO).

As shown in Figure 2, the IRAO algorithm is constructed by an iterated learning-based framework which can be separated into three stages: the offloading decision-making stage, the radio resource slicing stage and the algorithm parameters updating stage. The whole process, including the three stages, is named as step t, because they are executed recurrently in each time frame T indexed as t and we assume that t∈T={1,2…,T}. Step t is supposed to be completed after all the three stages are completed once and the learning-based algorithm will execute these three stages recurrently. Based on a DNN model, an offloading policy function $\pi :G,\Omega ,E\to {𝔁}^{*}$ is applied to estimate the optimal offloading decisions denoted by ${𝔁}^{*}=\{{𝓍}_{i}|{𝓍}_{i}\in \left\{0,1\right\},i\in I\}$. The performance of the policy π will be improved gradually with training step t increasing and it will reach a near-optimal level after sufficient training episodes EP.

### 4.2. Offloading Decision-Making

In the offloading decision-making stage, we design a priority estimation algorithm [12] applied a DNN based model with several layers and abundant neurons, which is described in detail in Section 5.2. At the beginning of each time frame t, the DNN employs parameters $\theta_{t}$ as the weights that connect the neurons and $\theta_{1}$ denotes the randomly initialized parameters which follow a zero-mean normal distribution. During each time frame t, the wireless channel gains are denoted by $G_{t}=\{G_{t,i}|i\in I,t\in T\}$ and the computational demand levels are denoted by $\Omega_{t}=\{\Omega_{t,i}|i\in I,\;\;t\in T\}$ and the available energy resources are denoted by $E_{t}=\{E_{t,i}|i\in I,t\in T\}$. Then, the DNN takes $G_{t}$, $\Omega_{t}$ and $E_{t}$ as input variables and outputs a vector containing a set of estimated priorities as

(8) $$\varepsilon_{t}=f_{\theta t}\left(G_{t},\Omega_{t},E_{t}\right)$$

where $\varepsilon_{t}=\{\varepsilon_{t,i}|i\in I,0\leq \varepsilon_{t,i}\leq 1\}$. In addition, some appropriate activation functions are used to modify the results derived from every layer in the DNN based model. 

With the estimated priorities $\varepsilon_{t}$, we generate K alternative binary offloading decisions $K{𝔁}_{t}=\{{𝔁}_{k,t}|k=1,2,\ldots ,K\}$ by the function

(9) $$K{𝔁}_{t}=f_{TH}\left(K,\;\varepsilon_{t}\right)$$

where K could be any integer within $\left[1,2^{I}\right]$. In order to find a better solution to the decision ${𝔁}_{t}$, a larger K should be set to enlarge the exploration space. However, it will take more time to execute the algorithm. Thus, the trade-off between optimality and complexity should be balanced and we apply a dynamic threshold-iterated quantization method which could generate at most  K alternative binary offloading decisions. In detail, these decisions ${𝔁}_{k,t}$ are generated as follows. 

Firstly, we set a default threshold $TH_{0}$ which is used for comparison with the elements $\varepsilon_{t,i}$ in $\varepsilon_{t}$. Secondly, we calculate the distances between the elements $\varepsilon_{t,i}$ and the presetting threshold $TH_{0}$, then the elements $\varepsilon_{t,i}$ are sorted by these distances from smallest to largest. Thirdly, the dynamic threshold $TH_{k,t}$ is iteratively set as the sorted elements $\varepsilon_{t,i}$, respectively. We created K binary offloading decisions ${𝔁}_{k,t}=\{{𝔁}_{k,t,i}|i\in I\}$ correspondingly with the following function

(10) $${𝓍}_{k,t,i}=\{1,\;if\;\varepsilon_{t,i}>TH_{k,t};\;0,\;if\;\varepsilon_{t,i}\leq TH_{k,t}\}$$

where k=1,…,K. Finally, these K alternative decisions ${𝔁}_{k,t}$ are input to the next time frame slicing part and then K values $U3^{*}\left(G_{t},\Omega_{t},E_{t},{𝔁}_{k,t}\right)$ are calculated correspondingly. Accordingly, the optimal offloading decision ${𝔁}_{k,t}{}^{*}$ with the maximized $Q3^{*}$ is selected by the function,

(11) $${𝔁}_{k,t}{}^{*}=arg\underset{{𝔁}_{k,t}}{\;max}\;U3^{*}\left(G_{t},\Omega_{t},E_{t},{𝔁}_{k,t}\right)$$

where **𝔁**_k,t_^*^ ∈ {**𝔁**_*k,t*_|*k* = 1, …, 𝒦}.

### 4.3. Radio Resource Slicing 

In the frame slicing stage, we slice each time frame t for different user devices i with the set $\tau_{t}=\{\tau_{t,i}|i\in I,0\leq \tau_{t,i}\leq 1\}$, subject to $\sum_{i=1}^{I}\tau_{t,i}+\lambda_{t,i}\leq 1$. In order to find the optimal slicing decision set $\tau_{t}{}^{*}$, we formulate it as a convex problem $U3^{*}\left(G_{t},\Omega_{t},E_{t},{𝔁}_{k,t}\right)$, which is described in Section 3. We use a one-dimensional bi-section search algorithm over a dual variable which is presented in references [10,12,16]. As these 𝒦 binary offloading decisions **𝔁**_*k,t*_ are derived in the offloading decision- making stage, the slicing decisions are generated by the function,

(12) $$\tau_{k,t}=f\left(G_{t},\Omega_{t},E_{t},{𝔁}_{k,t}\right)=arg\;\underset{\tau }{\max}\;U\left(G_{t},\Omega_{t},E_{t},{𝔁}_{k,t},\tau \right)$$

Subsequently, we calculate and compare the K utilities $U\left(G_{t},\Omega_{t},E_{t},{𝔁}_{k,t},\;\tau_{k,t}\right)$. With the best utilities $U^{*}\left(G_{t},\Omega_{t},E_{t},{𝔁}_{k,t},\;\tau_{k,t}\right)$, we can obtain the optimal decisions $\left\{{𝔁}_{k,t}{}^{*},\tau_{k,t}{}^{*}\right\}$ correspondingly. 

### 4.4. Algorithm Parameters Updating

In the algorithm parameters updating stage, an experience replay technique is applied. We get the optimal decision ${𝔁}_{k,t}{}^{*}$ at each step t and store the most recent experience denoted by tuple ($G_{t},\Omega_{t},E_{t},{𝔁}_{k,t}{}^{*})$ in a replay memory set ${𝓜}_{t}$ correspondingly. At the beginning of the algorithm, the set ${𝓜}_{t}$ is initially empty with a limited size $\left|{𝓜}_{t}\right|$. Considering the importance sampling trick, the oldest tuple will be popped if the cache of set ${𝓜}_{t}$ overflows. We denote the index of time frame by m and the memory set is given by

(13) $${𝓜}_{t}=\{\left(G_{m},\Omega_{m},E_{m},{𝔁}_{m}\right)|m\epsilon \left[\sigma ,\ldots ,t-1,t\right]\}$$

where $\left\{\sigma =0,\;if\;t\leq \left|{𝓜}_{t}\right|;\;\sigma =t-\left|{𝓜}_{t}\right|,\;if\;t>\left|{𝓜}_{t}\right|\right\}$.

In order to enhance the training efficiency of the algorithm, the DNN based model is not trained in every step t. Thus, we set a training episode between several steps with an appropriate training interval δ, which means that the training process is executed once an episode. In every training episode, an experience replay sampling method is applied for reducing the complexity of using entire tuples in set ${𝓜}_{t}$. In detail, we randomly extract a mini-batch of experience tuples from the memory set ${𝓜}_{t}$ and store them in the empty training buffer set ${𝓑}_{t}$ with a size of $\left|{𝓑}_{t}\right|$. The time frame index of sampled tuple is denoted by b and the set of these indexes is denoted by β. The sampled buffer set is given by

(14) $${𝓑}_{t}=\{\left(G_{b},\Omega_{b},E_{b},\;{𝔁}_{b}\right)|b\epsilon \beta \}$$

Subsequently, the extracted tuples are used as labeled samples and the DNN based model is trained by minimizing the cross-entropy loss with an optimizer algorithm denoted as $f_{opt}\left(\theta_{t}\right)$, such as Adam and RMSprop. We use an estimated value $f_{t,b}\;\mathrm{caclulated}\;\mathrm{by}\;f_{\theta t}\left(G_{b},\Omega_{b},E_{b}\right)$ and define a loss function $L\left(\theta_{t}\right)$ of mean value sigmoid cross-entropy as

(15) $$L\left(\theta_{t}\right)=\frac{-1}{\left|{𝓑}_{t}\right|}\sum_{b\epsilon \beta }\left({𝔁}_{b}\ln f_{t,b}+\left(1-{𝔁}_{b}\right)\ln\left(1-f_{t,b}\right)\right)$$

Then, the parameters of the DNN based model are updated from $\theta_{t}$ to $\theta_{t+1}$ with a befitting parameter named learning rate lr. Accordingly, the policy $\pi_{\theta t}$ is updated to a new policy $\pi_{\theta t+1}$ after the training process. For convenience of expression, the model is named as the DNN model in the following, which is trained by a single sampled buffer once per episode.

**Figure 3:**
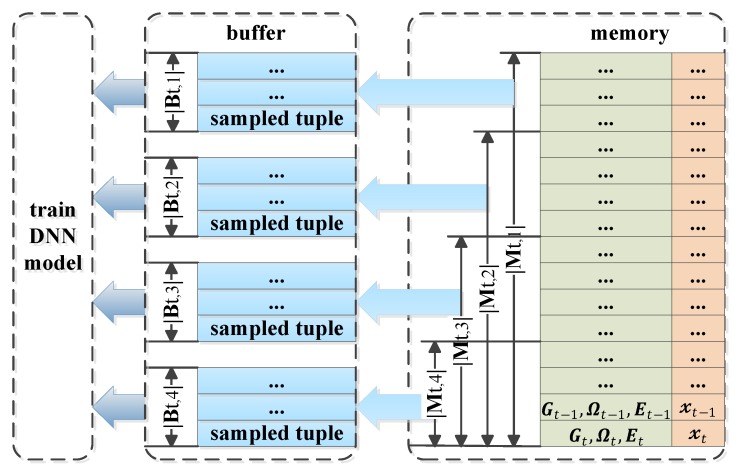
Training process of asynchronously trained deep neural network (AsyDNN) model.

Moreover, in order to enhance the algorithm performance in terms of training speed and estimating accuracy, we modify the DNN model with some tricks. At first, we design the synchronously trained DNN model named the SynDNN model, which is trained by the same synchronously sampled buffer set ${𝓑}_{t}$ several times with a relatively smaller learning rate lr in each training episode. This trick could accelerate the learning speed but still not reduce the possibility of getting into the local optimum caused by over-fitting, which is evaluated in Section 5. Then, referring to the distributed sampling trick, which is applied in an RL-based asynchronous advantage actor-critic (A3C) algorithm, we design an asynchronously trained DNN model named the AsyDNN model. As shown in Figure 3, it adopts several different replay memories ${𝓜}_{t,\alpha }$ and is trained by asynchronously sampled buffer sets given by

(16) $${𝓑}_{t,\alpha }=\{\left(G_{b},\Omega_{b},E_{b},\;{𝔁}_{b}\right)|b\epsilon \beta_{\alpha }\}$$

where α represents the index of buffer number. Especially, the sizes $\left|{𝓜}_{t,\alpha }\right|$ of different memories are set distinctively, referring to the trick of prioritized experience replay in an RL-based algorithm. Obviously, this AsyDNN model could improve the training performance by reducing the sampling bias and the performance is compared with that of the DNN model and the SynDNN model in Section 5.

### 4.5. Algorithm Iteration

With regard to the parameter K, the algorithm could speed up with a smaller K as explained previously in Section 4.2. As the DNN based model becomes more accurate after some training episodes, the number K could be reduced gradually. Number K is initialized as *I* and it is reduced along with $k_{t}$ which denotes the ordered index of the optimal decision ${𝔁}_{k,t}{}^{*}$ in all calculated decisions ${𝔁}_{k,t}$. In order to eliminate fluctuation of the number K and maintain the performance of the DNN based model, an empirical value parameter ρ is set as the update interval steps of K, that is K is updated as 

(17) $$K=min\left(1+max\left\{k_{i}|i=t-1,\ldots ,t-\rho \;\right\},\;I\right)$$

The algorithm is executed iteratively according to these processes. In this way, the performance results of the algorithm gradually become stable and converge into good values after sufficient training episodes. Thus, we denote $EP_{max}$ as the number of training episodes and correspondingly the maximum number of steps is set as $T=\delta *EP_{max}$, that is, 1≤t≤T. For a better explanation, the pseudo code of the IRAO algorithm is shown in Algorithm 1. 

**Algorithm1:** Intelligent Rapid Adaptive Offloading Algorithm1:Initialize the DNN models with random parameters $\theta_{1}$, memory size $\left|{𝓜}_{t}\right|$, $\left|{𝓜}_{t,\alpha }\right|$, buffer size $\left|{𝓑}_{t,\alpha }\right|$, training interval δ and learning rate lr;2:t = 1;3:For step $t\leq \delta *EP_{max}$:4: Input $G_{t}$, $\Omega_{t}$ and;5: Generate estimated priorities $\varepsilon_{t}$ by $\varepsilon_{t}=f_{\theta t}\left(G_{t},\Omega_{t},E_{t}\right)$;6: Reset K, *TH*;7: Derive K sets decisions $K{𝔁}_{t}$ by $K{𝔁}_{t}=f_{TH}\left(K,\;\varepsilon_{t}\right)$;8: Obtain K utilities $U3^{*}\left(G_{t},\Omega_{t},E_{t},{𝔁}_{k,t}\right)=\underset{\tau }{\;max}\;U\left(G_{t},\Omega_{t},E_{t},{𝔁}_{k,t},\tau \right)$;9: Select optimal decision ${𝔁}_{k,t}{}^{*}=arg\underset{k}{\;max}\;U3^{*}\left(G_{t},\Omega_{t},E_{t},{𝔁}_{k,t}\right)$;10: Output the optimal decision ${𝔁}_{k,t}{}^{*}$;11: Store the tuple $\left(G_{t},\Omega_{t},E_{t},{𝔁}_{k,t}{}^{*}\right)$ into experience memory ${𝓜}_{t}$;12: If t % δ == 0:13:  While Asy == True:14:   Extract mini-batch tuples $\left\{\left(G_{b},\Omega_{b},E_{b},\;{𝔁}_{b}\right)|b\epsilon \beta_{\alpha }\right\}$ from ${𝓜}_{t,\alpha }$;15:   Store these tuples in several different buffer sets ${𝓑}_{t,\alpha }$;16:   Minimize the loss $L\left(\theta_{t}\right)$ by Adam optimizer iteratively;17:  Parameters $\theta_{t}$ are updated to $\theta_{t+1}$;18:  Policy $\pi_{\theta t}$ is updated to a new policy $\pi_{\theta t+1}$;19: *t* = *t* +1;20:Store the DNN model with the optimized parameters $\theta_{t}$.

## 5. Simulation and Evaluation

In this section, numerical simulations are presented to evaluate the performance of the proposed IRAO algorithm in a dynamic IoT system. Firstly, the simulations environment is introduced and the related parameters are presented. Secondly, we tune algorithm variables and hyper parameters of the DNN based model and we investigate the influence of them. Thirdly, different convergence processes of three DNN based models are illustrated. Fourthly, some simulations are conducted with different numbers of devices, which show that the proposed algorithm has good scalability. Fifthly, the strategies generated from the IRAO are near-optimal compared with those generated from one baseline algorithm, which outperform those of the other candidates. Finally, we evaluate the algorithm efficiency of different algorithms. 

### 5.1. System Parameters Setup

As shown in Table 2, we set some suitable values to the relevant parameters of the system after some investigations in practice. In our simulations of the multi-user IoT system, the default number of users is set as I=10, that is, i∈{1,…,10}. We assume that the devices possess same hardware features and the computing energy efficiency coefficient is given by $e_{i}=10^{-27}$ and the data bit process speed is given by ∅=100(cycle/bit). Considering the dynamic characteristics of service requirements, the computational service demand levels are randomly set as 0.5 or 1, that is, $\Omega_{t}=\{\Omega_{t,i}|\Omega_{t,i}\in \left\{0.5,\;1\right\},i\in I]$. In view of signaling load and interference noise, we assume the bandwidth utility ratio $v_{u}=1.2$ and the channel noise power $N=10^{-9}W$. Without loss of generality, the time frame is set as T=1s.

We assume the wireless communication channel applies the Industrial Scientific Medical (ISM) frequency band based on 802.11 protocol. Accordingly, the communicating carrier frequency is set as *f_c_* = 2450 MHz and the channel bandwidth is set as *W* = 52 MHz. Considering that all accessed IoT devices are in the small coverage area of a wireless network, we assume the distances between user devices and the AP to be $d_{i}\in \left[10,30\right]$ (m). Without loss of generality, these distances follow a uniform distribution and are set from a small value to a big value as the number *i* increases. Besides, *λ_c_* denotes the carrier wavelength and *c* denotes the speed of electromagnetic wave. With the free space path loss model given by

(18) $$L{\left(path\right)}_{i}={\left(4\pi d_{i}/\lambda_{c}\right)}^{2}={\left(4\pi d_{i}f_{c}/c\right)}^{2}$$

the path losses can be expressed in decibels as $L{\left(path\right)}_{i}\left(dB\right)$ and calculated by $-28.5+20lgf_{c}\left(MHz\right)+20lgd_{i}\left(m\right)$. We assume the transmitting antenna power gain as $G_{T}=5\;dB$ and the receiving antenna power gain as $G_{R}=5\;dB$. The average channel power gain is given by 

(19) $${\bar{G}}_{i}\left(dB\right)=G_{T}+G_{R}-L{\left(path\right)}_{i}\left(dB\right)$$

**Figure 4:**
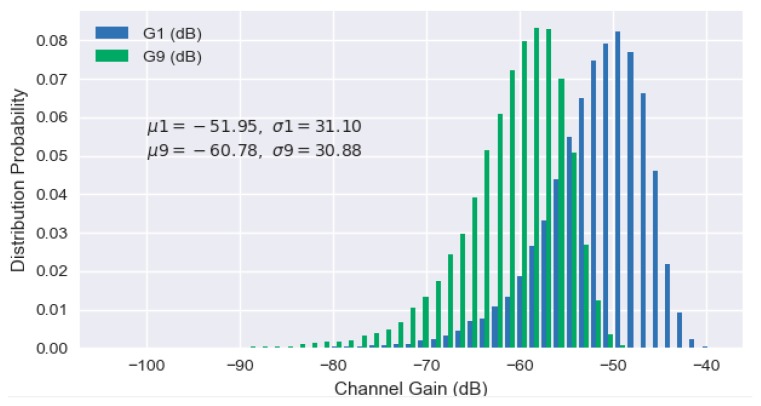
Frequency distribution histogram of different channel power gains.

Due to the factor $d_{i}$, ${\bar{G}}_{i}$ decrease as the number i increases. During each time frame *t*, each channel power gain is assumed to remain the same as a quasi-static value, which could be expressed as $G_{i,t}={\bar{G}}_{i}\alpha_{i,t}$ with an independent channel fading factor $\alpha_{i,t}$. In different time frames, the channel power gains are assumed to be time-varying with the factor $\alpha_{i,t}$ which is a dynamic variable following a specific distribution. For a better explanation, we plot the frequency distribution histogram of channel power gains $G_{1}$ and $G_{9}$ for user 1 and user 9, respectively, which follow the Rayleigh distribution as shown in Figure 4.

### 5.2. Algorithm Parameters Tuning

For improving the performance of the IRAO algorithm, we conduct simulations to explore the fitting values for related parameters. In our simulations, all the experiments are conducted on a computer with Intel Quad Core i5-4590 CPU @ 3.3 GHz and 4 GB RAM and the algorithm is implemented by Python 3.6. Tensor Flow 1.0 is used to construct the DNN models.

**Figure 5:**
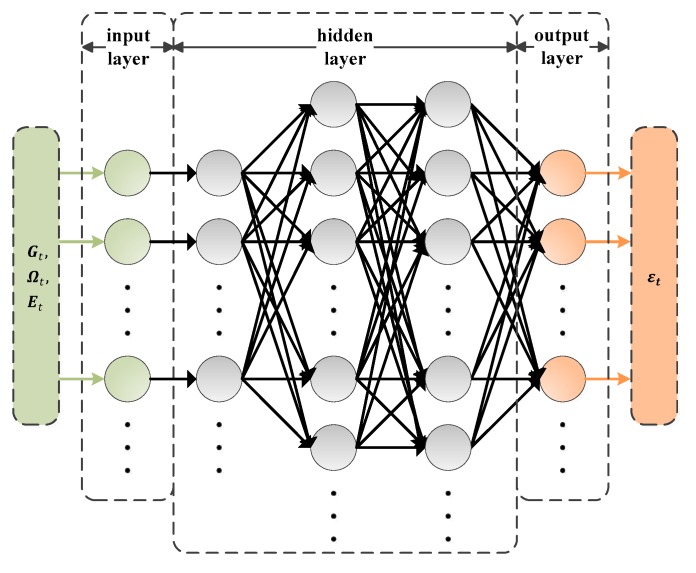
Designed architecture of DNN.

To implement the priority estimation algorithm, the DNN based model is firstly designed by default with a five-layer connected architecture, that is the input layer, the three hidden layers and the output layer, as shown in Figure 5. The input layer consists of 30 neurons which are used to input the information about wireless channel gains, computational demand levels and available energy resources. In the first of the three hidden layers, a kernel function is firstly applied to transfer the input variables with a non-linear feature, which uses a Gaussian function as the activation function. In the other two hidden layers, a ReLU function is used as the activation function to modify the middle values, which is denoted by $f_{Relu}\left(𝓍\right)=max\left(0,\;𝓍\right)$ and the number of neurons are 90 and 80, respectively. We set 10 neurons in the output layer and a sigmoid function is used to bound the final results denoted by $f{\left(𝓍\right)}_{sigmoid}=1/\left(1+e^{-x}\right)$.

For each training episode, the SynDNN model is trained four times with a smaller learning rate which is a quarter of that in the DNN model and the AsyDNN model is trained by four different buffer sets ${𝓑}_{t,\alpha }$ accordingly. The Adam estimation method is used as the optimizer $f_{opt}\left(\theta_{t}\right)$ by default for updating the parameters $\theta_{t}$ of the DNN based models. Furthermore, the frame slicing problem is solved by several standard optimization methods integrated in SciPy library. 

In our simulations, we set the overall steps as T=10,000 and we separate them into two parts, that is, the steps for the training process are set as T1=7000 and the steps for testing process are set as T2=3000. In order to evaluate the optimality of the best derived offloading decision ${𝔁}_{k,t}{}^{*}$, we normalized the corresponding utilities $U^{*}\left(G_{t},\Omega_{t},E_{t},{𝔁}_{k,t}{}^{*}\right)$ as the normalized utilities $U_{nor}\left(G_{t},\Omega_{t},E_{t},{𝔁}_{k,t}{}^{*}\right)$, that is,

(20) $$U_{nor}\left(G_{t},\Omega_{t},E_{t},{𝔁}_{k,t}{}^{*}\right)=U^{*}\left(G_{t},\Omega_{t},E_{t},{𝔁}_{k,t}{}^{*}\right)/U^{*}\left(G_{t},\Omega_{t},E_{t}\right)$$

Therein, the baseline utilities $U^{*}\left(G_{t},\Omega_{t},E_{t}\right)$ are calculated by a coordinate descent adaptive offloading (CDAO) algorithm extended from the CD iterative search algorithm [13,16] and bisection search algorithm [10,12] which are mentioned in Section 2. The CDAO algorithm exploits a similar radio resource slicing methods to IRAO and applies the CD method to search offloading decisions, which has been investigated and shown to be able to obtain near optimal solutions in related works. We also consider an exhaustive adaptive offloading (EAO) algorithm which has a similar radio resource slicing method to IRAO but applies an exhaustive method to select an optimal offloading decision. In other words, the candidate binary offloading decisions in EAO are enumerated for all possible solutions to the number of $2^{I}$. It is easy to infer that the execution time of the EAO algorithm consumes too much time, because the optimal binary offloading decisions are selected by iterated operations among all $2^{I}$ possible candidate solutions which cause the curse of dimensionality. 

**Figure 6:**
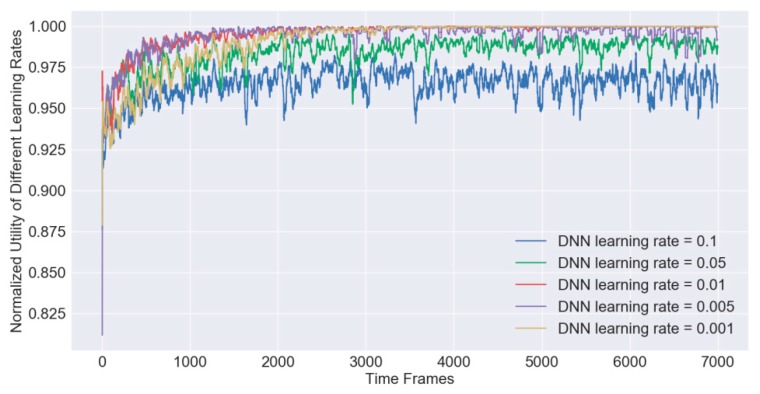
The IRAO training processes of different learning rates.

In order to obtain a fitting hyper parameters of the DNN based models for the IRAO algorithm, extensive simulations are conducted under different hyper parameters, such as memory size $\left|{𝓜}_{t}\right|$, batch size $\left|{𝓑}_{𝓉}\right|$, learning rate lr, training interval δ and update interval ρ. For example, under different learning rates, we plot the dynamic learning curves which are formed by the moving average normalized utilities $U_{nor}\left(G_{t},\Omega_{t},E_{t},{𝔁}_{k,t}{}^{*}\right)$ by a window of 30 as shown in Figure 6. In other words, each point in the figure denotes the average value of 30 utilities, which is the same for the following figures in this paper. It shows that the moving average utilities $U_{nor}\left(G_{t},\Omega_{t},E_{t},{𝔁}_{k,t}{}^{*}\right)$ gradually converge to the value 1 as the training step t increase and the performance of the algorithm becomes relatively stable after 2000 iterative steps. When the larger learning rate parameter is applied, it will get a faster convergence speed but suffer from a larger possibility of getting into a local optimal solution. With the empirical value derived after extensive simulations, we set the learning rate parameter as lr=0.01. Similarly, we find other fitting parameters as follows, $|{𝓜}_{t}|=1024$, $\left|{𝓑}_{t}\right|=256$, $|{𝓜}_{t,1}|=1024$, $|{𝓜}_{t,2}|=896$, $|{𝓜}_{t,3}|=768$, $|{𝓜}_{t,4}|=640$, $\left|{𝓑}_{t,\alpha }\right|=256$, δ=10 and ρ=32. We apply them as the default values in the rest of the simulations.

### 5.3. DNN Models Selection

**Figure 7:**
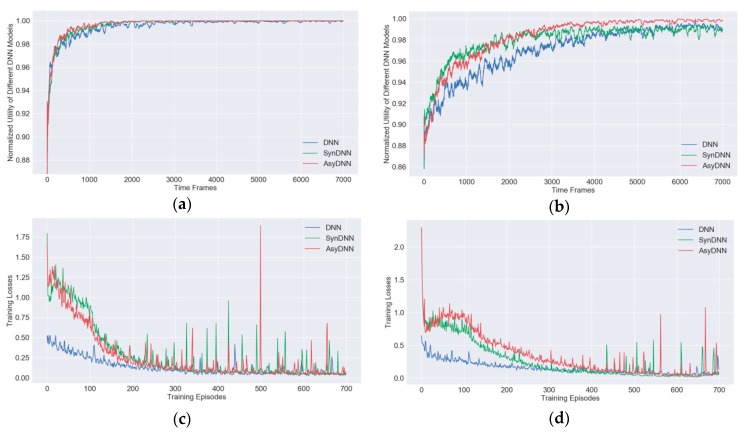
Training processes and training losses of the IRAO algorithm with different DNN models: (**a**) the training processes under 10 users; (**b**) the training processes under 20 users; (**c**) the training losses under 10 users; (**d**) the training losses under 20 users.

For getting a better training convergence performance of IRAO, some simulations are conducted to compare the training process of different DNN models. Figure 7a,b show that our proposed IRAO can generate near-optimal offloading decisions, as the normalized utilities $U_{nor}\left(G_{t},\Omega_{t},E_{t},{𝔁}_{k,t}{}^{*}\right)$ converge to the value 1. Compared with the DNN model and the SynDNN model, the normalized utilities of the AsyDNN model have a faster convergence rate and obtain better stabilized values especially when the number of users is large as shown in Figure 7b. Therefore, it is apparent that the IRAO algorithm with the AsyDNN model would have a better scalability. This is further analyzed in Section 5.4.

Meanwhile, during the training episodes, the training losses $L\left(\theta_{t}\right)$ of different DNN training models are shown in Figure 7c,d. It shows that the training losses of the SynDNN model and the AsyDNN model are larger than those of the DNN model at the beginning of the training processes. With steps increasing, the losses of both the SynDNN model and the AsyDNN model converge to a small value close to that of the DNN model. In particular, the losses of the AsyDNN model decrease faster than those of the SynDNN model.

### 5.4. Algorithm Scalability Analyses

**Figure 8:**
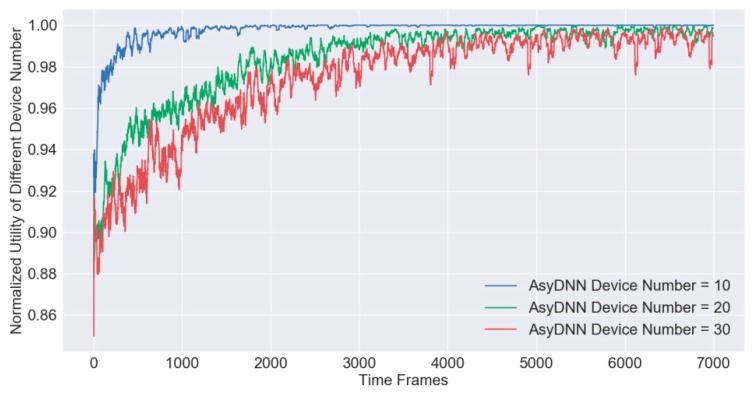
Scalability analyses of training processes for the IRAO algorithm.

For testing the algorithm performance of scalability, we conduct simulations under different numbers of access devices and plot the moving average curves as shown in Figure 8. At first, an offline training process with 7000 steps is accomplished and a trained AsyDNN model with fitting parameters is generated. Afterwards, a quick booting process with the model is applied for the online testing process in the next 3000 steps, which is similar to the process of online increment learning. In detail, the hotbooting technique initializes the parameters according to the model trained in similar environments rather than initializing them randomly, which can accelerate the learning speed and improve the model accuracy [27]. After calculating the normalized utility $U_{nor}\left(G_{t},\Omega_{t},{𝔁}_{k,t}{}^{*}\right)$ under 10, 20 and 30 devices, separately, the results illustrate that all AsyDNN models have a good convergence performance in terms of stability and optimality. The mean value of normalized utilities is still above 0.97 even if there are 30 devices, which proves that the scalability of the IRAO algorithm is excellent.

### 5.5. Algorithm Effectiveness Comparison

**Figure 9:**
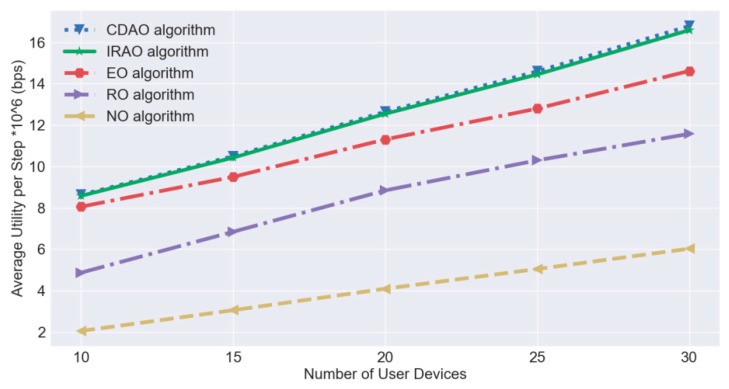
Algorithm effectiveness comparison of the IRAO and candidates.

For quantizing the effectiveness of our proposed IRAO algorithm in the testing process, we calculate the average value of utilities by weighted sum computing rates in the unit of bits per second. Meanwhile, we compare it with the other four baseline algorithms including the CDAO algorithm—which is described in Section 5.2—the entire offloading (EO) algorithm, the random offloading (RO) algorithm and the non-offloading (NO) algorithm. The EO and RO algorithms exploit a similar radio resource slicing method to IRAO as well but each device entirely offloads computational services to the edge server in EO and each device randomly offloads computational services to the edge server in RO. As for the NO algorithm, all computational services are executed locally on each user device itself. As shown in Figure 9, the average utility increases as the number of IoT devices increase. When there are 30 devices, the average utility is above 16 Mbps. Moreover, the performance of the proposed IRAO is close to that of CDAO which is near optimal and they all clearly outperform other three baseline algorithms.

### 5.6. Algorithm Efficiency Evaluation

**Figure 10:**
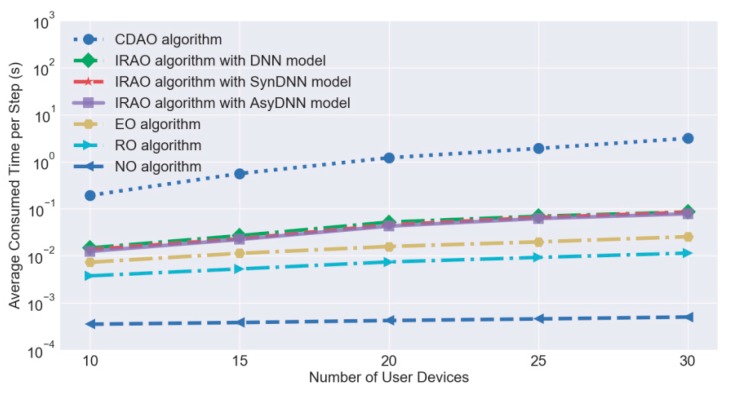
Algorithm efficiency evaluation of IRAO and candidates.

Some other simulations are conducted to test the execution time for evaluating algorithm efficiency. As the EAO algorithm consumes too much time, the CDAO algorithm is proposed for obtaining near-optimal utilities without high algorithm complexity. However, the CDAO algorithm still consumes too much time for real-time controlling due to the nature of iterated operations. As the DNN based algorithm can reduce the number of alternative offloading decisions by a fitting predictive model, the efficiency of the offloading decision-making algorithm can be further improved without compromising much performance of optimality. For better evaluation, we calculate the average execution time per step consumed by these comparable algorithms including the CDAO algorithm, the proposed IRAO algorithms with different models, EO algorithm, RO algorithm and NO algorithm. Figure 10, with a logarithmic ordinate, presents the consumed time in seconds and it shows that all IRAO algorithms take remarkable shorter time than the CDAO algorithm which is ten times longer in general. Further, the execution time of the CDAO algorithm clearly increases with the number of accessed devices, which still poses a dimensionality problem to large scale networks. As our proposed IRAO algorithms can make the decisions within 0.1 s for 30 devices, it is possible to achieve real-time control in rapidly changing environments.

## 6. Conclusions

As restricted resources have seriously limited the computational performance of massive IoT devices and industrial sensors, better processing capability is urgently required. In this paper, we consider a time-varying IoT system consisting of multiple devices with different computational requirements and wireless channels. In order to improve the performance of computational services in all participants, an IRAO algorithm is proposed for an IoT system based on an MEC network. Through a learning-based framework, it can continually derive adaptive strategies combining offloading decision making with radio resource slicing. In particular, the binary offloading policies are generated by a priority estimation algorithm, which is designed based on a DNN model trained with replaying experiences. To improve the optimality of the IRAO, extensive numerical simulations are conducted to explore the relationship between algorithm performance and variable parameters. For example, the DNN model has a good convergence performance when the learning rate is set to 0.01. For further improving the optimality and convergence rate of DNN model, its training process is refined and new models are name as the SynDNN model and the AsyDNN model. Some simulations are conducted to analyze the algorithm scalability when the numbers of the devices are 10, 20 and 30. Other simulations demonstrate that the IRAO algorithm has excellent effectiveness and efficiency when it is compared with other candidate algorithms. On our simulation platform, the proposed IRAO algorithm with the AsyDNN model can derive near-optimal and real-time strategies in less than 0.1 second even if the system has 30 devices. In the foreseeable future, the proposed algorithm will be amended to further improve the performance under different constraints in diverse scenarios. It will also be extended with different radio resource slicing methods and additional computational resource allocation methods. More experiments will be conducted to verify the algorithm performance under real platforms and innovative network architectures [31].

## Figures and Tables

**Table 1:** The comparisons of related works and our work.

Work	System Model	Optimization Objective	Constraint Condition	Generated Strategy	Applied Method
[10]	multi-user, multi-server	task completion time, energy consumption	transmission power border, wireless channel, finite computing resource	task offloading, transmission power, computing resource	bisection search, heuristic
[12]	multi-user, single BS	weighted sum mobile energy consumption	infinite or finite cloud computation capacity, computation latency, channel qualities	offloading priority, radio resource allocation	threshold-based bisection search
[13]	multi-user, single BS	total mobile-energy consumption	heterogeneous arrival instants and computation-deadlines	data partitioning for offloading, transmissions time division	block CD search
[16]	multi-user, single AP, integrated server	weighted sum computing rate	harvesting energy, dynamic radio resource, static service demand	computing offloading decisions, transmission time allocation	bisection search, CD, alternating direction
[26]	multi-BS, multi-server, multi-cache	received SNR, computation capability, cache state	networking, caching and computing resources	BS assigning, cache deciding, offloading deciding	double dueling DQN
[27]	single IoT device, multiple edge devices	energy consumption, computation latency, task drop rate	current battery level, previous radio transmission rate, predicted harvested energy	Edge devices selecting, offloading rate	CNN, RL
[28]	single mobile user, multi-BS, single server	execution delay, task drops, queuing delay, failing penalty, server payment	task queue state, channel qualities, energy queue state	task offloading, energy allocation	double DQN
[29]	multi-user, single edge server	energy consumption, task completion delay	static uplink bandwidth	offloading decisions, bandwidth allocation	parallel DNN
our work with IRAO	multi-user, single AP, virtualized server	weighted sum computing rate	available energy, dynamic radio resource, dynamic service demand	computing offloading decisions, radio resource slicing	asynchronous trained DNN

**Table 2:** The related symbols in our simulations.

Symbol	Definition	Setup
i	The number of user devices	i∈I={1,…,10}
$e_{i}$	computing energy efficiency	$10^{-27}$
∅	data bit process speed	100 (cycle/bit)
$\Omega_{t,i}$	computational service demand weight	$\Omega_{t,i}\in \left\{0.5,1\right\}$
$v_{u}$	bandwidth utility ratio	1.2
N	channel noise power	$10^{-9\;}W$
T	time frame	1s
$f_{c}$	communicating carrier frequency	2450 MHz
W	channel bandwidth	20 MHz
$d_{i}$	distance between user i and AP	$d_{i}\in \left[10,30\right]\;\left(meters\right)$
c	speed of electromagnetic wave	$3*10^{8}m/s$
$L{\left(path\right)}_{i}$	free space path loss	${\left(4\pi d_{i}f_{c}/c\right)}^{2}$
$G_{T}$	transmitting antenna gain	5dB
$G_{R}$	receiving antenna gain	5dB
${\bar{G}}_{i}$	average channel gain	$G_{T}G_{R}/L{\left(path\right)}_{i}\left(dB\right)$
$G_{t,i}$	each channel gain	${\bar{G}}_{i}\alpha_{i,t}$
t	the number of time frame	1≤t≤T=10,000
